# Risk factors for low birthweight in the public-hospitals at Peshawar, NWFP-Pakistan

**DOI:** 10.1186/1471-2458-8-197

**Published:** 2008-06-04

**Authors:** Sareer Badshah, Linda Mason, Kenneth McKelvie, Roger Payne, Paulo JG Lisboa

**Affiliations:** 1 Deprtment of Statistics, Islamia College, University of Peshawar, Pakistan; 2 School of Applied Community and Social Studies, Liverpool John Moores University, Liverpool, UK; 3 Formerly School of Computing and Mathematical Sciences, Liverpool John Moores University Liverpool, UK; 4 Biomathematics and Bioinformatics Department, Rothamsted Research, Harpenden, Herts, AL5 2JQ, UK; 5 School of Computing and Mathematical Sciences, Liverpool John Moores University Liverpool, UK

## Abstract

**Background:**

Low birthweight is a widely used indicator of newborn health. This study investigates the association of birthweight <2.5 kg (LBW) with a wide range of factors related to geo-demographics, maternal health and pregnancy history in public hospitals at Peshawar, North West Frontier Province (NWFP) Pakistan. It is noted that that Low birthweight may arise for two different reasons, one related to gestational age and the other corresponding to births that are small for gestational age (SGA).

**Methods:**

Data on geo-demographics, maternal health indicators, pregnancy history and outcome scores for newborn babies and their families (*n *= 1039) were collected prospectively between August and November 2003 in a cross-sectional survey of four public hospitals in Peshawar, NWFP-Pakistan. Crude and adjusted odds ratios were used to investigate the factors affecting incidence of LBW, by multivariate logistic regression. Gestational age was included as an explanatory variable therefore the additional covariates identified by model selection are expected to account for SGA.

**Results:**

The main geo-demographic risk factors for SGA identified in this study, controlling for gestational age of less than 37 weeks, are maternal age, nationality and consanguinity. Presentation with anaemia and the history of previous abortion/miscarriage were also found to be significant independent factors. The adjusted odds ratio for gestational age showed the largest effect in explaining the incidence of LBW. The next highest odds ratio was for maternal age below 20 years. The explanatory model included two pairwise interactions, for which the predicted incidence figures for LBW show an increase among the Tribal area with presentation of anaemia, and among full term babies with their mothers having a previous history of abortion/miscarriage.

**Conclusion:**

In addition to gestational age, specific factors related to geo-demographics (maternal age, consanguinity and nationality), maternal health (anaemia) and pregnancy history (abortion/miscarriage) were significantly associated with the incidence of LBW observed at the four hospitals surveyed in Peshawar. These results indicate that cultural factors can adversely affect the incidence of SGA in this area of Pakistan.

## Background

Across the world, neonatal mortality is 20 times more likely for low birthweight (LBW) babies compared to heavier babies (≤ 2.5 kg)[1]. It is also established as an important risk factor for neonatal morbidity [2,3]. The cohort of LBW (birthweight <2.5 kg) babies is likely to reflect two effects, namely a short gestational age (preterm births) and small for gestational age (SGA). Small for gestational age usually results from intra-uterine growth restriction (IUGR) [4]. However, if the mother is small, it may be normal for her to have a small fetus. In the current study gestational age is included as an explanatory variable, therefore the study is focused on the identification of risk factors for the complementary effect which is SGA.

In a literature survey, de Onis et al (1998) found that IUGR babies are at increased risk of perinatal mortality and morbidity, i.e. sudden infant death syndrome, poor cognitive development and neurologic impairment, cardiovascular disease, high blood pressure, obstructive lung disease, diabetes, high cholesterol concentrations and renal damage in adulthood [4]. Such babies remain a burden on government expense in developed countries and a permanent problem for their families in developing countries.

The incidence of LBW (<2.5 kg irrespective of gestational age) is estimated to be 16% worldwide, 19% in the least developed and developing countries and 7% in the developed countries. The incidence of LBW is 31% in South Asia followed by Middle East and North Africa (15%), Sub-Saharan Africa (14%) and East Asia and Pacific 7% [5]. Of the total estimated IUGR babies (<2.5 kg and = >37 weeks), Asia accounts for 75%, and with 20% and 5% born in Africa and Latin America, respectively. The IUGR accounts for 11% of the total babies in developing countries ranging from 2% to 21%, that is 6 times higher compared to developed countries [4].

In South Asia the incidence of LBW is 36%, 30% in Bangladesh and India, and 19% in Pakistan [5]. In Pakistan the LBW rate varies from 5% to 23% in different parts [6-11], whilst IUGR in a community-based study in Karachi was found to be 24.4% [12].

Peshawar is the capital of the NWFP Province of Pakistan, where the health care facilities are used by the people of Settled areas of NWFP, and Federally Administered Tribal Areas of Pakistan (FATA or Tribal areas) including Afghan refugees since 1979. The diversity in area-status (Tribal/Settled), ethnicity (Afghan refugees/Pakistani) and differential in overall geo-demographics factors suggest a need to investigate LBW in Peshawar. The current study derives an explanatory multivariate regression model for LBW, that includes gestational age as an independent covariate. The remaining selected covariates are thus interpreted as explanatory of incidence of LBW under the category of SGA. Therefore, this prospective public hospital-based study in Peshawar focuses on LBW to investigate associated explanatory factors beyond gestational age. These factors contribute towards the explanation of the observed births that are SGA (including preterm and non-preterm SGA births).

## Methods

Data were collected in a cross-sectional prospective survey on maternal and paternal geo-demographic factors, maternal health and pregnancy history (MHPH) and neonatal outcome from all public hospitals (Hayatabad Medical Complex, Khyber Teaching Hospital, Lady Reading Hospital, and Government Maternity Hospital) in Peshawar during August to November 2003 through clinicians on duty.

The research reported in the paper was approved by the Research Ethics Committee of Liverpool John Moores University, with reference number 02141. Approval was granted prior to the commencement of data collection by questionnaire. Verbal consent was obtained from each mother recruited for this study.

The data comprise questionnaire responses collected from 1,039 single birth mothers from a total of 2286 mothers delivered during the study period irrespective of birth-status and gestational age. The volunteer clinicians for this study collected data as per their usual shifts i.e. day time one week and night time the next. This gave us 45.5% of the available data. As far as possible all consecutive births during the duty periods were recruited into this study, resulting in only 4.5% records returned entirely empty. This may have been through refusal by the patient to take part in the study or an inadvertent omission on the part of the duty clinician.

Women were interviewed by the clinicians in local languages at admission in the reception, with the exception of emergency admissions, when they were interviewed in the labour room. The factors were recorded on a pre-designed questionnaire validated by health professionals during a pilot study in the same hospitals.

Five factors were collected as continuous measures, which were later banded into categorical measures according to the previous literature (Table 1 and 2). These measures include maternal age [14-16], gestational age [16,17], height [12,18] and the gap between this and the previous pregnancy [19]. However, the threshold for maternal pregnancy weight during analysis was chosen as 57 kg for this study, as this value had the highest significance in the univariate analysis for LBW. For the purpose of this paper consanguinity was included under geo-demographic factors (Table 1). Pregnancy registration was taken as proxy for pre-natal care.

**Table 1:** Univariate analysis of geo-demographic maternal risk factors for LBW in Peshawar, 2003.

Variable		Birthweight		OR	95% CI
		<2.5 kg	≥ 2.5 kg		
	(N = 1039)	(n = 101)	(n = 938)		
**Area of residence**					
Tribal area	243(24.0)	34(14.0)	209(86.0)	1.7	[1.1, 2.7] *
Settled area	770(76.0)	66(8.6)	704(91.4)		
**Water sources**					
Non-fresh	373(36.8)	49(13.1)	324(86.9)	1.8	[1.2, 2.7] **
Fresh	641(63.2)	51(8.0)	590(92.0)		
**Nationality**					
Afghan refugees	120(11.7)	23(19.2)	97(80.8)	2.5	[1.5, 4.2] **
Local people	903(88.3)	78(8.6)	825(91.4)		
**Consanguinity**					
Consanguineous	611(60.0)	73(11.9)	538(88.1)	2.0	[1.3, 3.2] **
Non-Consanguineous	407(40.0)	26(6.4)	381(93.6)		
**Maternal age**					
<20 years	77(7.5)	25(32.5)	52(67.5)	6.1	[3.6, 10.7] **
>34 years	172(16.8)	20(11.4)	152(88.4)	1.7	[1.0, 2.9]
20–34 years	772(75.6)	56(7.3)	716(92.7)		
**Family income**					
<5000 rupees	701(69.4)	80(11.4)	621(88.6)	1.8	[1.1, 2.9] *
≥ 5000 rupees	309(30.6)	21(6.8)	288(93.2)		
**Maternel education**					
Illiterate	708(69.5)	81(11.4)	627(88.6)	2.1	[1.2, 3.6] **
Non-illiterate	310(30.5)	18(5.8)	292(94.2)		
**Paternal education**					
Illiterate	382(37.6)	47(12.3)	335(87.7)	1.6	[1.1, 2.4] *
Non-illiterate	635(62.4)	52(8.2)	583(91.8)		

**p < 0.01, *p < 0.05

The category of LBW was defined as less than 2.5 kg [13]. Gestational age was calculated from the first day of the last menstrual period reported by the mother and categorised such that any delivery from 24 and <37 weeks were termed as preterm birth [13]. Some mothers may have had estimates of gestational age derived by ultrasound measurement made at antenatal visits, although this would not be the case for un-registered pregnancies which account for more than half of the observations.

**Figure 1:**
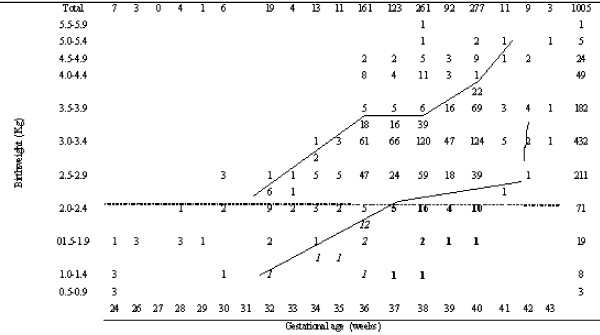
Distribution of birth weight versus gestational age.

The reliability of the estimates of gestational age was further investigated using a descriptive table showing the incidence of observations grouped by birthweight and gestational weeks, shown in the form of a normogram in Figure 1. This also show boundaries for the lower 10^th ^percentile (<10^th ^percentiles) and upper 10^th ^percentiles (>90^th ^percentiles) of birthweight.

A comparison of the profile of birthweight against gestational age with those listed in [20,21] shows a remarkable level of agreement on inspection of the two sets of data. This is interesting to note as one set of figures was derived in a technologically advanced city from an industrialised country, and the second comes from a single hospital study in Karachi, while our figures refer to four public hospitals in a border province of Pakistan. This consistency was taken to indicate that the estimates of gestational age in the prospectively acquired data set are sufficiently good to justify a description of the LBW cohort as shown in the Table, comprising low weight births expected for gestational age and SGA, the latter including a sub group of IUGR. The analysis shown in the next section takes gestational age as an independent variable, so seek to explain the residual variations due to SGA.

From the covariates originally collected, twenty were used for the initial analysis, to keep at least 5 events (LBW) per factor, as there are 101 occurrences of LBW in the data. This ratio of events per variable is on the threshold of acceptability for the size of the pool of covariates prior to initiating the variable selection process, as recommended in the literature [22].

There were two levels of data analysis, Crude odds of LBW babies in different categories were calculated to identify the significant factors at univariate level [23]. Multivariate logistic regression [24,25] was applied to uncorrelated groups of factors (Pearson's, Spearman's correlation with thresholds of 0.6), fitting the models using the software package SPSS [26] to assess the independent effects of factors on SGA [12] based on a backward stepwise approach. Wald's test was used for the significance of the factors in multivariate models [27] and chi-square was used for univariate models.

The interpretation of main effects and interactions was aided by calculating a one-way table of predicted means for each significant main effect, and a two-way table of predicted means for each significant (two-way) interaction, using the statistical package GenStat [28]. These were constructed by first forming a table containing fitted values for every combination of the seven factors in the model, then averaging over the factors that are not in the required table of predictions. This averaging was weighted using estimated population weights, formed by multiplying together a one-way table of weights for each factor, containing the proportions of cases recorded in each of its levels.

## Results

The results are for data from 1039 birth episodes, prospectively sampled in the four public hospitals in Peshawar. The overall incidence of LBW (<2.5 kg) was found to be 9.9%. However, the crude odds of LBW in all geo-demographic factors (i.e. area of residence, water sources, ethnicity, consanguinity, maternal age, family income, maternal and paternal education) (Table 1), and seven other factors from MHPH (i.e. gestational age, hypertension, anaemia, maternal pregnant weight, total abortion/miscarriage, abortion/miscarriage before this pregnancy, and maternal pregnancy registration), were found to be statistically significant at univariate level (Table 2).

**Table 2:** Univariate analysis of maternal health and pregnancy history, risk factors for LBW in Peshawar, 2003.

**Variable**		Birthweight		OR	95% CI
		<2.5 kg	≥ 2.5 kg		
	(N = 1039)	(n = 101)	(n = 938)		
**Gestational age**					
Preterm	229(22.8)	60(26.2)	169(73.8)	6.4	[4.1, 9.8] **
Full term	776(77.2)	41(5.3)	735(94.7)		
**Maternal diabetes**					
Yes	27(2.7)	3(11.1)	24(88.9)	1.2	[0.4, 3.9]
No	974(97.3)	96(9.9)	878(90.1)		
**Maternal hypertension**					
Yes	183(18.3)	25(13.7)	158(86.3)	1.6	[1.0, 2.6]*
No	816(81.7)	74(9.1)	742(90.9)		
**Maternal anaemia**					
Yes	383(38.5)	47(12.3)	336(87.7)	1.5	[1.0, 2.3] *
No	613(61.5)	52(8.5)	561(91.5)		
**Other health conditions**					
Yes	59(6.0)	8(13.6)	51(86.4)	1.5	[0.7, 3.3]
No	918(94.0)	87(9.5)	831(90.5)		
**Maternal height**					
<1.55 mtr	310(30.8)	38(12.3)	272(87.7)	1.4	[0.9, 2.2]
≥ 1.55 mtr	696(69.2)	62(8.9)	634(91.1)		
**Maternal weight**					
<57 kg	246(24.4)	35(14.2)	211(85.8)	1.8	[1.2, 2.8] **
≥ 57 kg	763(75.6)	65(8.5)	698(91.5)		
**Maternal BMI**					
≤ 19.0 kg/m^2^	40(4.0)	4(10)	36(90)	1.0	[0.4, 2.9]
>19.0 kg/m^2^	958(96.0)	96(10)	862(90)		
**Any abortion/miscarriage^a^**					
Yes	232(22.7)	33(14.2)	199(85.8)	1.8	[1.1, 2.7] *
No	788(77.3)	68(8.6)	720(91.4)		
**Abortion/miscarriage^b^**					
Yes	172(17.2)	25(14.5)	147(85.5)	1.7	[1.1, 2.8] *
No	826(82.8)	74(9.0)	752(91.0)		
**Gap between pregnancies^c^**					
<1.5 years	220(33.2)	25(11.4)	195(88.6)	1.1	[0.7, 1.9]
≥ 1.5 years	443(66.8)	46(10.4)	397(89.6)		
**Pregnancy registration**					
No	545(53.9)	67(12.3)	478(87.7)	1.8	[1.2, 2.8] **
Yes	467(46.1)	34(7.3)	433(92.7)		

**p < 0.01, *p < 0.05, BMI (body mass index), ^a ^History of any abortion/miscarriage in reproductive life, | ^b ^Abortion/miscarriage immediately preceding this pregnancy, ^c ^gap between this and the previous pregnancy (excluding primiparas mothers).

In the total LBW babies, 41.6% (42/101) were preterm and appropriate for gestational age (≥ 10^th ^percentile, AGA), 17.8 (18/101) were preterm and small for gestational age (<10^th ^percentile, SGA) and 40.6% (41/101) were full term and SGA. Among the total LBW, 58.4% (59/101) were SGA, and 40.6% (41/101) were appropriate for gestational age (Figure 1).

### Results from multivariate logistic regression

The adjusted odds of the SGA were also calculated and nine factors were found to be significant in different separate multivariable models adjusting for gestational age. Seven of these factors, namely area of residence, nationality, consanguinity, maternal age, gestational age, anaemia and abortion/miscarriage, were found to be significant risk factors for high incidence of SGA using stepwise backward logistic regression. Further analyses were carried out to identify potential interactions between the explanatory variables. This resulted in a definitive model with three geo-demographical indicators, nationality, consanguinity and maternal age at birth, and two pair-wise interactions respectively, i.e. (i) area of residence and history of anaemia, and (ii) gestational age and abortion/miscarriage (Table 3).

**Table 3:** Adjusted odds ratios and 95% confidence intervals for the final logistic regression model.

**Factors**	**B**	**AdjOR (95% CI)**	**PPr. of LBW**	**S. E**.
**Nationality**				
Afghan refugee	0.970	2.64 (1.39, 5.01) **	0.17	0.032
Local (Ref.)	-	-	0.09	0.009
**Consanguinity**				
Consanguineous	0.871	2.39 (1.39, 4.12)**	0.12	0.012
Non-consanguineous (Ref.)	-	-	0.06	0.012
**Maternal age**				
<20 years	2.122	8.35 (4.36, 15.98) **	0.32	0.048
>34 years	0.099	1.11(0.58, 2.11)	0.08	0.019
20–34 years (Ref.)	-	-	0.08	0.009

**Interactions**				
**Area × anaemia**				
[Tribal area] [Anaemia (yes)]	1.198	3.31(1.70, 6.50)**	0.20	0.035
[Tribal area] [Anaemia (no)]	-0.134	0.88(0.40, 1.90)	0.08	0.021
[Settled area] [Anaemia (yes)]	-0.106	0.90(0.49, 1.65)	0.08	0.015
[Settled area] [Anaemia (no)] (Ref.)	-	-	0.09	0.013
				
**Gestation × Abortion/miscarriage**				
[Preterm] [Abortion/miscarriage (yes)]	2.399	11.01(5.00, 24.23)**	0.25	0.052
[Preterm] [Abortion/miscarriage (no)]	2.350	10.49(5.73, 19.20)**	0.25	0.032
[Full term] [Abortion/miscarriage (yes)]	1.209	3.35(1.66, 6.77)**	0.11	0.023
[Full term] [Abortion/miscarriage (no)] (Ref.)		-	0.04	0.008
				
**Intercept**	-4.530**			

**p < 0.01, *p < 0.05, Ref. (Reference Category), AdjOR (Adjusted Odds Ratios), C.I (Confidence Interval), PPr. (the predicted probability of LBW from the model, or prediction for the probability of LBW in future), and S.E. (Standard Error of the predicted probability).

The adjusted odds ratios reinforce the earlier results from univariate analysis, showing that five factors significantly increase the incidence of LBW, namely Afghan refugees compared to Pakistani mothers, consanguineous compared to non-consanguineous births, and teenage compared to middle age mothers (Table 3). A further interactive term shows that anaemic mothers in Tribal areas are at increased risk of SGA compared to non-anaemic mothers in the Settled areas (Table 3). Moreover, the effect of abortion/miscarriage were seen in the case of full term babies, where the odds of SGA babied were 3.4 times higher compared to full term without abortion/miscarriages (Table 3).

The estimated predicted probability of LBW (PPr), obtained by marginalising over all of the explanatory variables, for single and interactive effects, respectively, are entirely consistent with the expectations from the values of the adjusted odds-ratios. The predictive analysis confirmed the significant independent effects already noted for teenage mothers, Afghan refugee mothers and consanguineous births, effect of anaemia in Tribal areas, and abortion/miscarriage on SGA (Table 3).

## Discussion

This study has been conducted in public hospitals that cover only 9% (urban = 18%, rural = 6%) of the total births, whereas the majority 91% of the deliveries take place at home (78%), or in private hospital/clinics etc (13%) [29]. There is no proper system that can record their history at home and due to the non availability of databases this study had to recruit patients prospectively from the four main public hospitals in Peshawar. This necessarily excludes births in private hospitals, clinics and at home. Due to limited voluntary participation of clinicians, it was not possible to collect information from all mothers admitted for delivery in the four hospitals from August to November.

In the present study abortion/miscarriage was used to include induced and spontaneous abortion, "due to the taboos and sensitivity associated with reporting an induced abortion"[30]. It was not possible to interview women at their homes in privacy to separate induced and spontaneous abortion. It should be noted that pregnancy registration was used as a proxy for prenatal care. We were also unable to collect data on energy intake that might be one of the important factors in Peshawar as reported by others in developing countries [31].

Considering the reliability of the study, this study found that some of the factors, i.e. consanguinity, low family income, maternal and paternal education and non-fresh water areas, diabetes, hypertension, anaemia and abortions are comparable with other reports for population or population-based studies. However, the high ratio of mothers from Tribal areas 24% in this study, compared to the total female Tribal population 15% [32] in this region (NWFP plus Tribal area) may over estimate, whereas, the low proportion of teenage mothers 7.5% compared to compared to 19.6% [19] may underestimate the incidence of LBW in this study.

The motivation behind this study is to collect data on low birthweight in NWFP, starting from the four public hospitals available for the general public in Peshawar. This study provides baseline information and a start to debate low birthweight from public hospitals in this region, which could help with possible intervention regarding maternal and newborn health in the future. It is an observational study which generated a sample surveying all but a very small proportion of consecutive births attended by the clinicians who agreed to take part. While this reduced the overall sample size, the duty patterns alternated between day and night and so the stratification of sampling by clinician does not indicate any factor likely to introduce bias into the results of the study.

The overall incidence of LBW in this study at Peshawar (10%) was half that of recent studies (19%–23%) in Lahore and Karachi [7,9] and the overall national average [5]. The differential in the incidence of LBW might be due to ethnicity/racial differences in Lahore and Karachi, compared to Peshawar. However, the incidence of LBW in Budhni village near Peshawar was reported to be 5% [6]. The variation in the present study and the Budhni village study could be due to the differences in the population based and hospital-based study. In the hospital-based study, the ratio of mothers at risk is suspected to be more prevalent than in a village study, due to referral of high risk mothers from the Basic Health Units (BHUs) based in villages. Another reason for the high incidence of LBW babies in our study compared to Northrop-Clewes study [6] could be the inclusion of mothers from Tribal areas and Afghan refugees. These mothers from Tribal areas and Afghan refugees were found to be at higher risk compared to mothers from Settled areas and Pakistani mothers, respectively.

Among the total LBW, 59% were SGA, and 41% appropriate for gestation (Figure 1). The main factors associated with SGA (maternal age, consanguinity, nationality, anaemia, and abortion/miscarriages) adjusting for gestational age are discussed as follows.

### Maternal age

Teenage mothers are well known for adverse pregnancy outcomes. However, in this study teenage mothers were independently associated with SGA compared to middle and older age mothers. The predicted probability of SGA was estimated to be 0.32, 0.08 and 0.08 for teenage, middle age and older age mothers, respectively (Table 3). Furthermore, we found that teenage mothers were independently associated with low maternal weight and had low family income (OR = 2.3, 1.8, p < 0.01) compared to the middle group of maternal age.

Instead of teenage mothers, studies in Karachi and Brazil associated maternal height, weight and primiparity [12,33], height, BMI and primiparity in Canada [34], and maternal weight and social status in Brazil and India [33,35,36]. Further studies on teenage mothers could be helpful in explaining the role of teenage mothers and its association with adverse pregnancy outcomes.

### Consanguinity

Consanguinity is common in developing countries due to social, cultural and economic reasons including traditions [19] (e.g. arranged marriages influenced by parents and near relatives especially uncles and the dilemma of preserving a pure blood-line etc.). To paraphrase a common cultural perception, "first and second cousin marriage is categorized as gold and silver, whereas non-consanguineous is considered worthless". That is why, in this study the majority of the people (60%) were found to be consanguineous, which is consistent with other community based studies in Pakistan [19,37].

In the present study, we found an independent effect of consanguinity on SGA. The impact of consanguinity in our study is consistent with other studies in Karachi [12], Pakistani Muslims in Birmingham UK [38] and the effect of genetic factors reported by Kramer in a review of adverse pregnancy outcomes [31].

### Afghan refugee status

The incidence of LBW in refugee camps varies from country to country. There is evidence that in the majority of the refugee camps the incidence of LBW is less than in their country of origin and their host country [42]. We found that Afghan refugee status increased the risk of SGA. We also found that Afghan refugee mothers were less likely to avail themselves of the health resources compared to Pakistani mothers (OR = 2.4, p < 0.01).

The higher incidence of adverse pregnancy outcome in Afghan refugee mothers is consistent with Vietnamese refugees in Hong Kong [38], refugees from Bosnia, Herzegovina and Serbia [40], but inconsistent with another study in Athens [41]. We suspect that the adverse outcomes in Afghan refugee mothers compared to local Pakistani mothers could be due to expected involvement of refugees in the Afghan-war that could affect pregnancy outcomes through mental health problems [43-46]. Further detailed study on Afghan refugee outcomes is recommended to understand the main causes of poor outcomes compared to Pakistani mothers.

### Abortion/miscarriages and anaemia

Abortion is well-known to be among the five leading causes of maternal mortality worldwide [47]. According to an estimate, approximately 150, 000 unwanted pregnancies are terminated each day worldwide by induced abortion, about 50,000 are terminated through unsafe abortion [30]. The estimated number of terminations/induced abortions is 890,000 annually in Pakistan, and nearly 200,000 women suffer from post-abortion complications [47]. Due to restrictive legal status in Pakistan [48], the majority of the miscarriages and induced abortions (80%) are attended by untrained birth attendants in unsafe conditions [49,50]. The main reason behind the high number of abortions could be a desire for small family size, unplanned pregnancy, sex selective abortions/gender preferences, or poverty [47].

**Figure 2:**
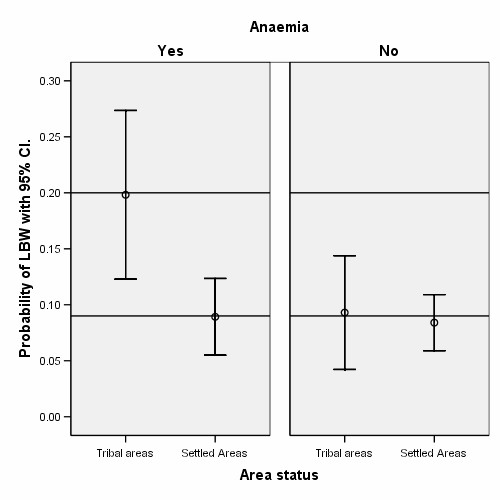
Distribution of LBW in Tribal and Settled areas with and without a history of anaemia during pregnancy, showing an interaction between the two explanatory variables.

In the present study, previous abortion/miscarriages were also associated independently with SGA babies (Table 3, Figure 2). It was further found that, the history of previous abortion/miscarriage were significantly higher in the hypertensive mothers during the present pregnancy than non-hypertensive mothers (OR = 1.9, p < 0.01) and mothers with the history of abortion/miscarriages were at increased risk of anaemia (OR = 1.5, p < 0.01) compared to mothers without history of abortion/miscarriage. The effect of previous abortions is consistent with another study in Ahmedabad [35], and other reports from developing countries [31].

**Figure 3:**
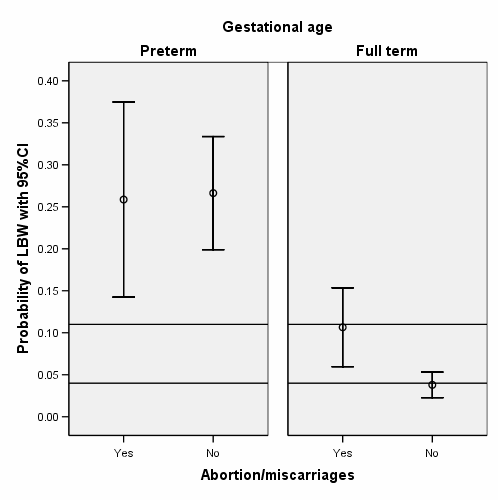
Distribution of LBW in preterm and full term with and without a history of previous abortion/miscarriages, indicating an effect for full-term births.

Anaemia is a common problem in developing countries in pregnant women, ranging from 8% to 33% in Pakistan [51], and increases the incidence of LBW and IUGR [17]. In the present study, anaemia was 38.5% and significantly higher in Tribal and deprived areas compared to Settled and developed areas (OR = 1.6, 2.0, p < 0.01). We found that anaemia was one of the main causes of SGA in the Tribal area compared to non-anaemic mothers in Tribal and Settled area (Table 3, Figure 3). The effect of anaemia in our study is consistent with other studies in Karachi and Ahmedabad [17,35].

This study also found that, full term babies whose mothers had a history of previous abortion/miscarriage were at higher risk (OR = 3.4, p < 0.01) compared to full term babies without any abortion/miscarriage (Table 3). Abortion affects maternal health through anaemia and haemorrhage [29], and pregnancy outcomes through low birthweight and short gestation [53]. In a hospital-based study in Calcutta-India Pahari et al [54] reported abortion as one of the main-causes of adverse pregnancy outcomes in addition to anaemia and hypertensive disorder.

## Conclusion

The incidence of low birthweight reported in this study is less than one-half of the overall figure for Pakistan. However, there is significant variation among groups of mothers with specific combinations of geo-demographic factors, maternal health indicators and pregnancy history.

The effect of anaemia and previous abortion/miscarriage in Tribal areas compared to Settled areas, and the independent effect of Afghan refugee status on birth weight need further investigation to identify the root causes of adverse pregnancy outcomes in Tribal areas and Afghan refugees.

Health awareness programmes delivered by health workers in the villages, at educational institutions and through a concerted media campaign regarding the effect of consanguinity, teenage mothers, abortions and anaemia could be helpful in reducing the adverse pregnancy outcomes.

The findings of this study are specific to public hospitals in NWFP-Pakistan. However they show value in registering with the hospital during pregnancy and potential value in improving data collection methods, perhaps by electronic means, to support the design of targeted public health interventions and monitoring of their effectiveness. Further studies on LBW in private hospitals and clinics, together with studies of home deliveries, are also needed in order to extend the scope of the present work on LBW in this region.

## Competing interests

The authors declare that they have no competing interests.

## Authors' contributions

SB carried out the survey, fitted the multivariate statistical model, and led the interpretation of the results and the writing of the paper. PJGL and KMcK provided supervision during the conception, design and statistical analysis. LM supervised the public health context of the work, during the conception and design of the survey as well as interpretation of the results. RP modelled the predictions using GENSTAT and verified the statistical integrity of the analysis. All authors read and approved the final version to be published.

## Pre-publication history

The pre-publication history for this paper can be accessed here:


